# Delayed cardiac tamponade caused by erosion 5 years after percutaneous closure of atrial septal defect with Figulla Flex II: a case report

**DOI:** 10.1093/jscr/rjaf104

**Published:** 2025-03-07

**Authors:** Kazuki Sakumoto, Yoshimori Araki, Mika Noda, Akihiro Kobayashi, Osamu Kawaguchi

**Affiliations:** Department of Cardiac Surgery, Toyota Kosei Hospital, 500-1 Ibobara Josui-cho, Toyota, Aichi 470-0396, Japan; Department of Cardiac Surgery, Toyota Kosei Hospital, 500-1 Ibobara Josui-cho, Toyota, Aichi 470-0396, Japan; Department of Cardiac Surgery, Toyota Kosei Hospital, 500-1 Ibobara Josui-cho, Toyota, Aichi 470-0396, Japan; Department of Cardiac Surgery, Toyota Kosei Hospital, 500-1 Ibobara Josui-cho, Toyota, Aichi 470-0396, Japan; Department of Cardiac Surgery, Toyota Kosei Hospital, 500-1 Ibobara Josui-cho, Toyota, Aichi 470-0396, Japan

**Keywords:** tamponade, erosion, percutaneous closure of atrial septal defect, Figulla Flex II

## Abstract

Percutaneous closure of atrial septal defect (ASD) is less invasive than surgical closure and yields good results; however, cardiac erosion is a serious complication. It usually occurs within 72 h after implantation and rarely after months to years. Cardiac erosion has been rarely reported using Figulla Flex II (FFII; Occlutech, Schaffhausen, Switzerland). In this case, we encountered a case of delayed cardiac erosion after FFII implantation. An 81-year-old woman, who underwent percutaneous ASD closure elsewhere 5 years previously, presented to our hospital with sudden chest pain and was diagnosed with cardiac tamponade. She underwent emergency surgery and was diagnosed with cardiac erosion caused by FFII. The device had penetrated the aortic wall through the right atrial wall. Removal and repair were successful. Cardiac erosion can occur long after implantation; therefore, patients require careful follow-up.

## Introduction

Percutaneous closure of atrial septal defect (ASD) was first reported in 1976 [1] and became widely used with the introduction of the Amplatzer Septal Occluder (ASO; Abbott Laboratories, Abbott Park, IL, USA) in 1997 [2]. Recently, Figulla Flex II (FFII; Occlutech, Schaffhausen, Switzerland) has been introduced and is a widely used alternative to ASO. Percutaneous closure of ASD is less invasive than surgical closure, has good results [3], and is recognized as the treatment of choice for secondary ASDs. Unfortunately, cardiac erosion is a serious complication, which is rare but fatal. It occurs within 72 h in most cases; however, it has rarely occurred a few months to years later [4]. In this report, we describe a case of cardiac erosion 5 years after FFII implantation which was successfully managed, saving the patient’s life.

## Case report

**Figure 1:**
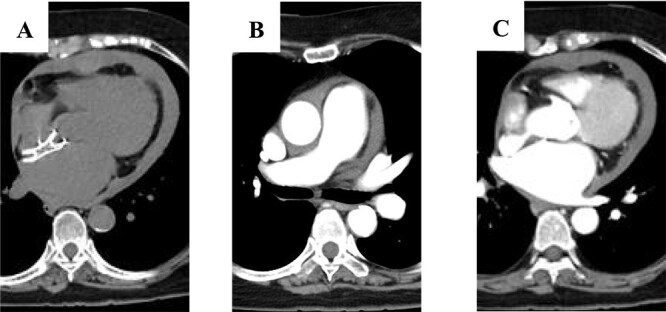
Computed tomography scan showing: (A) circumferential pericardial effusion; (B) no aortic dissection; and (C) no extravascular leakage of contrast media.

**Figure 2:**
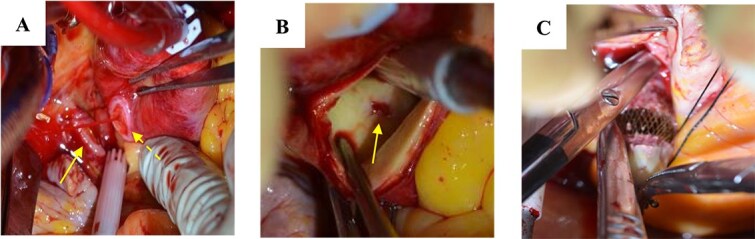
Surgical findings: (A) part of the device extruding from the right atrium (arrow) and a pinhole perforation on the aortic wall (dotted arrow); (B) perforation only without intimal injury inside the aorta; and (C) removal of the device.

An 81-year-old woman developed sudden chest pain after bathing and was admitted to our hospital. She had undergone percutaneous closure of ASD with FFII at another hospital 5 years previously. The patient was also receiving immunosuppressive drugs for rheumatoid arthritis. She was in shock on arrival. Computed tomography showed circumferential pericardial effusion without aortic dissection and extravascular leakage of the contrast medium (Fig. 1A–C). Emergency pericardial drainage was performed, and 450 mL of blood was drained. Although the cardiac tamponade was relieved, and the patient’s condition improved temporarily, blood drainage from the pericardial sac persisted. She was immediately transferred to the operating room for emergency surgery. We found that part of the device was exposed out of the right atrium, and a pinhole perforation with an ulcer was observed on the aortic wall adjacent to the exposed device; this was diagnosed as FFII-induced erosion (Fig. 2A). The aorta was obliquely incised to confirm that there was only a perforation without an intimal ulcer (Fig. 2B), the perforation was repaired with felt sandwich sutures, and the aorta was closed in two layers. The right atrium was then incised, and the device was easily removed by pulling the center pole and folding the device (Fig. 2C). The ASD and right atrial injury were closed with a bovine pericardial patch. The patient had a favorable postoperative course and was discharged without any major complications.

## Discussion

Although ASO has shown good results, one recognized complication is erosion. The literature reports that erosion causes half of all ASO-related deaths [5]. The mortality rate for percutaneous closure of ASD is low (0.093%), but it rises for emergency surgery for adverse events after percutaneous closure of ASD (2.6%) [5]; therefore, careful follow-up is necessary after device implantation.

ASO is a rigid device, whereas FFII has a flexible design. Because of its structure, FFII is often chosen for cases at a high risk for erosion. Risk factors for cardiac erosion include implantation of an oversized device compared to the diameter of the ASD, deficient aortic rim, and atrial septal malalignment [6]. In the present case, FFII was selected owing to a deficient aortic rim. The patient’s requirement for immunosuppressive drugs may have also contributed to the erosion.

Previous reports have indicated that in addition to erosive injury to the right and left atria, there could also be invasion of the aorta [7]. Aortic dissection has been previously reported; therefore, it is essential to ensure that the aorta is intact [8]. In the present case, an incision was made, and the aorta was checked from the inside. However, there was no tear, so an artificial vascular graft replacement could be avoided.

In conclusion, cardiac erosion can occur over a long period of time; therefore, careful follow-up is necessary after implantation and should be shared among institutions. When cardiac tamponade is observed in a patient after percutaneous closure of ASD, erosion should be strongly suspected, and prompt emergency surgery should be considered.
